# Albumin-adjusted calcium concentration should not be used to identify hypocalcaemia in critical illness

**DOI:** 10.1186/cc12384

**Published:** 2013-03-19

**Authors:** T Steele, R Kolamunnage-Dona, C Downey, C Toh, I Welters

**Affiliations:** 1 University of Liverpool, UK; 2 Royal Liverpool University Hospital, Liverpool, UK

## Introduction

Hypocalcaemia is common in critical illness and accurate assessment is crucial. Small studies have shown that albumin-adjusted calcium (adjCa) does not accurately predict the ionised calcium (iCa) concentration in critically ill patients, yet adjCa continues to be widely used [1]. We investigated the reliability of using adjCa to identify hypocalcaemia in a large, diverse population requiring intensive care.

## Methods

In a retrospective study of patients admitted to the ICUs of a tertiary care hospital between January 2008 and 2012, iCa and pH were extracted from routine blood gas results and total calcium, albumin and phosphate from routine biochemistry results. adjCa was calculated using a formula derived from and validated on the local population [2]. Sensitivity, specificity, positive and negative predictive values (PPV and NPV) and area under the curve (AUC) of adjCa for predicting hypocalcaemia (iCa <1.1 mmol/l) were calculated.

## Results

Data from 1,038 patients were included. Admission iCa was available for 976 (94%) patients. A total of 539 (55%) were hypocalcaemic (iCa <1.1 mmol/l). adjCa was available for 1,031 (99%), with 602 (58%) classified as hypocalcaemic (adjCa <2.2 mmol/l). adjCa <2.2 mmol/l had sensitivity of 78% and specificity of 63% for detecting iCa <1.1 mmol/l. The best previously published formula had an AUC of 0.81 (95% CI = 0.78 to 0.83), compared with an AUC of 0.78 (0.75 to 0.81) for the locally validated formula [1]. It was hypothesized that albumin, pH or phosphate derangements could affect the proportion of albumin-bound calcium and the performance of adjustment formulae. Albumin concentration was not correlated with the difference between adjCa and iCa. Weak but statistically significant correlations were found for pH (*r *= 0.164; *P *0.001) and phosphate (*r *= 0.135; *P *0.001). The sensitivity and specificity of adjCa <2.2 mmol/l for detecting hypocalcaemia did not improve when looking at patients with normal pH (sensitivity 60.4%; specificity 69%) or phosphate (32.2%; 78.9%).

## Conclusion

adjCa is a relatively poor predictor of iCa in critically ill patients. This cannot be explained by abnormalities of albumin, phosphate or pH. With direct iCa measurements now readily available on ward-based analysers, adjCa should not be used to determine calcium status in critical illness.
